# Protective, Anti-Inflammatory, and Anti-Aging Effects of Soy Isoflavones on Skin Cells: An Overview of In Vitro and In Vivo Studies

**DOI:** 10.3390/molecules29235790

**Published:** 2024-12-07

**Authors:** Magdalena Wójciak, Piotr Drozdowski, Agnieszka Skalska-Kamińska, Martyna Zagórska-Dziok, Aleksandra Ziemlewska, Zofia Nizioł-Łukaszewska, Małgorzata Latalska

**Affiliations:** 1 Department of Analytical Chemistry, Medical University of Lublin, Chodźki 4a, 20-093 Lublin, Poland; agnieszka.skalska-kaminska@umlub.pl; 2 Department of Plastic Surgery, Specialist Medical Centre, 57-320 Polanica-Zdrój, Poland; 3 Department of Technology of Cosmetic and Pharmaceutical Products, Medical College, University of Information Technology and Management in Rzeszow, Sucharskiego 2, 35-225 Rzeszow, Poland; mazagorska@wsiz.edu.pl (M.Z.-D.); aziemlewska@wsiz.edu.pl (A.Z.); zniziol@wsiz.edu.pl (Z.N.-Ł.); 4 Department of General and Pediatric Ophthalmology, Medical University of Lublin, 20-079 Lublin, Poland; mlatalska@gmail.com

**Keywords:** phytoestrogens, topical application, genistein, daidzein, fibroblasts, keratinocytes, skin

## Abstract

Isoflavones are found in numerous plant species within the Leguminosae family; however, soy isoflavones are particularly significant in practice and have been extensively studied in recent years. The health-promoting potential of orally administered soy isoflavones is widely documented in the scientific literature, and many review articles have been developed to highlight their significance. However, it should be noted that soy-isoflavone-rich extracts and isolated soy isoflavones, such as genistein and daidzein, are also often applied topically as ingredients in many formulations, including face creams, tonics, and emulsions. New delivery systems are continuously being developed to enhance the skin permeability of isoflavones, thus improving their efficacy. In this context, their direct activity on skin cells is an important aspect of scientific research. The anti-inflammatory, protective, and antioxidant properties of isoflavones and soy extracts make them promising cosmetic ingredients with anti-aging potential because inflammation and the accumulation of reactive oxygen species (ROS) can lead to structural and functional changes in skin cells, accelerating the aging process. This review provides an overview of research on the impact of the application of soy isoflavone extract and soy-derived isoflavones on skin cells, with a focus on the documented molecular mechanisms underlying their effects. This study aims to offer essential insights to aid in the development of functional cosmetics and future clinical applications.

## 1. Introduction

**Figure 1:**
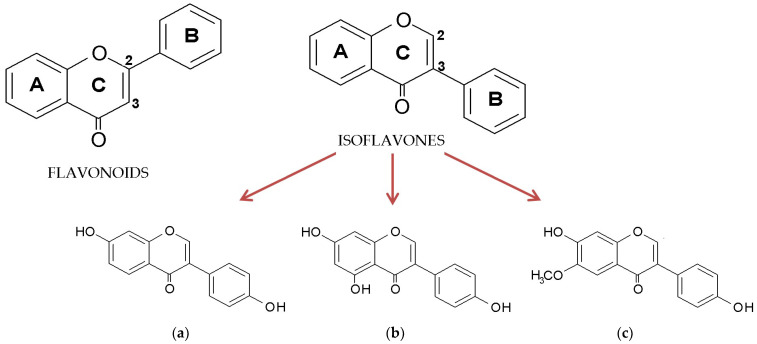
General structure of flavonoids and isoflavones and the chemical structure of aglycone forms of soy isoflavones: (**a**) daidzein, (**b**) genistein, and (**c**) glycitein.

Isoflavones belong to polyphenolic compounds and are distinguished from the flavonoid class based on the characteristic location of the second benzene ring attached to the heterocyclic pyran. In contrast to flavonoids, where the benzene ring (B) is attached to the pyran at position 2, in isoflavones, it is attached at position 3 (Figure 1) [1]. They have been found in numerous plant species belonging to the Leguminosae family, including soy (*Glycine max*), kudzu (*Pueraria lobata*), and red clover (*Trifolium pratense*). Many compounds from this subclass are quite well-known; however, soy isoflavones have the greatest significance in practice and have been intensively studied in recent years [2,3,4,5,6,7]. Soy contains mostly derivatives of genistein, daidzein, and glycitein, with the glycosides and malonylglycosides of these aglycones being the predominant forms (Figure 1a–c) [8].

The total isoflavone content may vary significantly, by up to three to four times, as their accumulation depends on the soy variety, the growing region, the season, and environmental conditions. Isoflavones are found mostly in the aerial parts of the plant, with the greatest abundance in the seeds [8].

**Figure 2:**
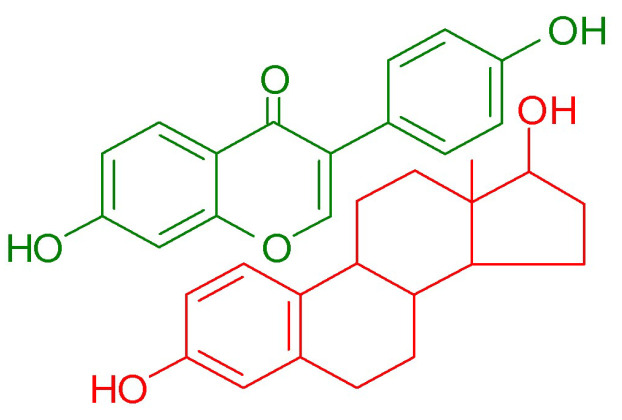
Comparison of chemical structure of daidzein (green) and 17β-estradiol (red).

The activity of isoflavones depends significantly on their glycosylation status. The conjugated forms, which are naturally present in soy, are generally less active compared to their aglycone counterparts. However, upon ingestion, isoflavone glycosides undergo hydrolysis by glucosidase enzymes in the gut. This process removes the sugar moiety, converting the glycosides into aglycones. These aglycones are further metabolized by intestinal microbiota, leading to the production of metabolites, such as equol and O-desmethylangolensin [1]. The biological properties of isoflavones have been the subject of extensive in vitro and in vivo investigations, and the health-promoting potential of orally administered soy isoflavones is widely described in numerous scientific papers. Studies have shown that they exhibit multidirectional biological activity, helping to prevent obesity [9], lower blood glucose levels [10], and reduce the risk of osteoporosis [11], certain types of cancer [12], and metabolic-related cardiovascular disorders [13]. Moreover, due to their structural similarity to 17β-estradiol (Figure 2), isoflavones can bind to estrogen receptors (ERs), including both ERα and ERβ, with a significantly higher affinity for the ERβ isoforms, which are predominantly found in the brain, bones, bladder, skin, lungs, and vascular endothelium. Isoflavones exert both estrogen agonist and antagonist effects depending on the tissue type, concentration, and hormonal environment [14]. In cases of estrogen deficiency, they exhibit mild estrogenic activity, which is leveraged in hormone replacement therapy during menopause to alleviate symptoms, such as hot flashes, night sweats, mood swings, and bone density loss [15]. Clinical data indicate that isoflavone consumption during menopause provides protection against cardiovascular disease, reduces the severity of osteoporosis, and improves cognitive function [16].

The majority of scientific papers are devoted to the beneficial effects of the internal use of isoflavones, and many review articles have been published on this topic [4,5,6,17,18]. However, it should be noted that extracts rich in soy isoflavones or isolated soy isoflavones are also used as ingredients in externally applied products, including face creams, tonics, and emulsions. New delivery systems, such as nanoemulsions and nanoemulsion-based hydrogels, are continuously being developed to enhance the skin permeability of isoflavones, thereby maximizing the effectiveness of these compounds [19,20,21,22]. The anti-inflammatory, protective, and antioxidant activities of isoflavones and soy extract make them promising cosmetic additives with anti-aging potential. Inflammation and the accumulation of reactive oxygen species (ROS) can lead to structural and functional alterations in skin cells. Therefore, reducing inflammation and preventing cellular oxidation may help slow down the aging process [23].

This review paper provides an overview of research on the impact of soy isoflavone extracts and soy-derived isoflavones on skin cells, with a focus on the molecular mechanisms involved in their activity. This study aims to provide essential insights into the topical application of isoflavones that could assist in the development of functional cosmetics and help plan future research directions.

A literature survey was conducted using the Scopus, PubMed, ScienceDirect, Web of Science, Springer, and Google Scholar databases. The following keywords were used in the investigations: “genistein”, “daidzein”, “glycitein”, “genistin”, “daidzin”, “glycitin”, or “soy” combined with “skin”, “fibroblast”, and “keratinocytes”. We selected all articles with available full text in English. The studies in which isoflavones were administered orally were excluded from the review.

## 2. Mechanisms of Action and Signaling Pathways Activated by Isoflavones in the Skin

**Figure 3:**
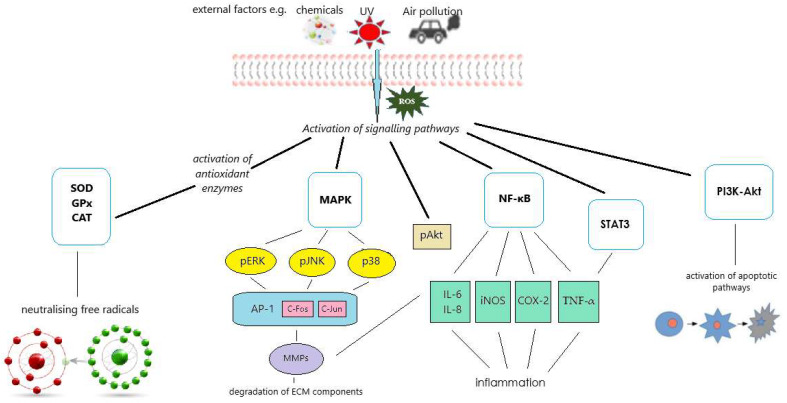
Diagram illustrating the molecular mechanisms studied for the protective effects of isoflavones on skin cells exposed to external stimuli. Reactive oxygen species (ROS) activate signaling pathways, including nuclear factor kappa B (NF-κB), phosphoinositide 3-kinase-protein kinase B (PI3K-Akt), signal transducer and activator of transcription 3 (STAT3), and mitogen-activated protein kinases (MAPK). These pathways can further induce inflammatory responses and promote the degradation of extracellular matrix (ECM) components, thereby contributing to skin damage and aging. In addition, ROS activate antioxidant enzyme systems, including catalase (CAT), superoxide dismutase (SOD), and glutathione peroxidase (GPx), which help mitigate oxidative stress and protect skin cells from damage.

Literature data indicate that isoflavones can contribute to skin health and anti-aging effects through several signaling pathways, including ROS/NF-κB, ROS/Akt/NF-κB, PI3K-Akt, STAT3, and MAPK [24,25,26,27,28,29]. The activation of these pathways via different external agents, including UVB and environmental pollutions, leads to the modulation of cellular responses and may result in inflammation, oxidative stress, apoptosis, and extracellular matrix remodeling (Figure 3).

NF-κB (nuclear factor kappa-light-chain-enhancer of activated B cells) is a crucial transcriptional regulator of the inflammatory response primarily found in the cytoplasm as a p65/p50 heterodimeric complex in its inactive form and bound to the inhibitory subunit IκBα. Activation of the catalytically active kinases IKKα/β, triggered by various mitogens, leads to the phosphorylation of IκBα, resulting in the release of free NF-κB. This free NF-κB translocates to the nucleus and initiates the transcription of various molecules, including cytokines, chemokines, cell adhesion molecules, and regulators of apoptosis and proliferation. Thus, phosphorylation of NF-κB p65 induced by inflammatory agents or by reactive oxygen species (ROS) triggers transcriptional activity and plays a crucial role in cell growth, proliferation, and survival [30,31]. STAT3 (Signal Transducer and Activator of Transcription 3) is another transcription factor that regulates the expression of target genes involved in inflammation, apoptosis, and tissue repair. STAT3 is considered a potential therapeutic target in disorders linked to chronic inflammation. Therefore, the STAT3–NF-κB as a classical signal transduction pathway activated by numerous cytokines and growth factors plays a critical role in regulating various cellular processes, including inflammation, cell survival, and immune responses [32].

The PI3K-Akt signaling pathway begins with the phosphorylation of phosphatidylinositol 4,5-bisphosphate (PIP2) by phosphoinositide 3-kinase (PI3K) to form phosphatidylinositol 3,4,5-trisphosphate (PIP3). PIP3 recruits and activates a serine/threonine kinase (Akt), which, upon activation, promotes cell survival by inhibiting apoptotic pathways, enhances protein synthesis, and regulates various metabolic processes [33].

In turn, the mitogen-activated protein kinase (MAPK) pathway consists of a series of protein kinases that transmit signals from cell surface receptors to the nucleus, resulting in specific cellular responses. The three primary MAPK proteins are the extracellular signal-regulated kinase (ERK), the c-Jun N-terminal kinase (JNK), and the p38 MAPK. Activation of MAPK typically occurs through a three-tiered kinase cascade involving a MAPK kinase kinase (MAPKKK), a MAPK kinase (MAPKK), and the MAPK itself [34].

The role of the transforming growth factor-beta (TGF-β)/Smad signaling pathway in regulating collagen synthesis has also been studied in relation to isoflavones [35]. TGF-β activates Smad proteins, which then translocate to the nucleus to promote the transcription of collagen genes, particularly type I and III collagen. This pathway stimulates collagen production and regulates the expression of enzymes involved in extracellular matrix remodeling, ensuring a balanced synthesis and degradation of collagen [36]. Additionally, it has been found that isoflavones affect the cell cycle through cyclin-dependent kinases (CDKs) [37]. CDKs are critical regulators of the cell cycle, ensuring that cells progress through the different phases (G1, S, G2, and M). When CDKs bind to specific cyclins, they become activated and phosphorylate target proteins that drive the cell through checkpoints, enabling DNA replication, repair, and division [38].

Furthermore, some studies examine the impact of soy isoflavones on the activity of antioxidant enzymes, including superoxide dismutase (SOD), glutathione peroxidase (GPx), and catalase (CAT) [25,39,40]. These enzymes are critical in neutralizing reactive oxygen species (ROS), contributing to antioxidant protection and mitigating cellular damage from UV radiation (photoprotection). SOD catalyzes the dismutation of superoxide radicals into oxygen and hydrogen peroxide, GPx reduces hydrogen peroxide to water, and CAT turns hydrogen peroxide into water and oxygen [41].

## 3. Protective Effect Against UV-Radiation-Induced Skin Damage

Exposure to UV radiation, especially at wavelengths ranging from 280 to 320 nm (UVB), causes unfavorable visible skin changes, including erythema, hyperpigmentation, hyperplasia, inflammation, and an increase in the level of wrinkles as a result of trans-epidermal water loss (TEWL), desquamation, and destruction of collagen fibers [42,43]. These effects are associated with a number of molecular processes, such as the activation of the epidermal growth factor receptor (EGFR), which leads to an increase in the thickness of the epidermis, the expression of matrix metalloproteinases, which are responsible for collagen decomposition, and the activation of cyclooxygenase-2 (COX-2), an enzyme that acts in the inflammatory response [44,45]. Moreover, under UV irradiation, excessive production of reactive oxygen species (ROS) occurs, which results in a decrease in the levels of antioxidant enzymes. This disruption of redox balance promotes unfavorable skin alterations and accelerated skin aging because ROS can accumulate, leading to the oxidation of cellular components, damage to structural elements, such as collagen fibers, inflammation, and an increased risk of skin diseases, including cancer [46]. Therefore, the search for new compounds that counteract these effects is important, as they could help protect the skin from the harmful effects of UV radiation, delay the photo-aging process, and reduce the risk of skin-related disorders.

Several studies, both in vivo and in vitro, have demonstrated the protective effect of soy isoflavones on the skin cells against the pathophysiological processes induced by UV radiation [24,25,47,48,49,50,51]. In vitro assays were conducted using two types of cell models, human keratinocytes (HaCaT cells) and human skin fibroblasts (BJ-5ta cells), which were irradiated with UVB at doses ranging from 20 to 70 mJ/cm^2^, focusing on both isoflavone mixtures and pure compounds.

An extensive study of the UVB protective activity of isoflavones was conducted by Iovine et al. [47]. They investigated the influence of genistein and daidzein on the expression of Gadd45, a gene involved in DNA repair that is induced upon DNA damage, and COX-2, which is responsible for edema, epidermal hyperplasia, inflammation, and carcinogenesis. They showed that the aglycones used individually did not alter or even increase the levels of Gadd45 and COX-2 in UVB-induced cells; however, this effect occurred without cytotoxicity. In turn, genistein and daidzein worked synergistically, and the combination of both compounds demonstrated a stronger photoprotective effect than the individual isoflavones. The mixture of genistein and daidzein contributed to the activation of DNA repair mechanisms by enhancing Gadd45 expression and acted as an anti-inflammatory agent by reducing COX-2 expression in cells exposed to UVB irradiation; however, this effect strongly depended on the concentration of isoflavones. Furthermore, Iovine et al. [48] conducted similar research using an RPH–aglycone mixture (soy extract titrated to 90% in isoflavone aglycones) and glucoside derivatives of genistein (genistin) and daidzein (daidzin). The most effective inhibition of UVB-induced expression of COX-2 and Gadd45 was noted for the combination of genistin and daidzin (2:2 µM) and for the isoflavone mixture at concentrations of 8–10 µM. The protective action of isoflavones against UVB-induced DNA damage was also evidenced by the comet assay [47,48]. The most important findings of Iovine et al. are summarized in Table 1.

The protective effect of genistein against UVB-induced DNA damage was also demonstrated by Moore et al., who investigated the levels of pyrimidine dimers (PDs)—photoproducts with cytotoxic and mutagenic activity—and the expression of proliferating cell nuclear antigen (PCNA), a marker of DNA repair. In reconstituted human skin subjected to UV radiation, genistein preserved the expression of PCNA, which was diminished after UVB treatment, and reduced PD formation [49]. In turn, Tang et al. observed a reduction in certain proinflammatory cytokines, including interleukin-1 (IL-1), macrophage migration inhibitory factor (MIF), and plasminogen activator inhibitor (PLANH1) released by UVB-stimulated keratinocytes [50].

Huang et al. investigated the photoprotective activity of soy aglycones and found that UVB-induced H_2_O_2_ levels in the HaCaT cell line were significantly reduced in the presence of genistein and daidzein (with daidzein being more effective), while no activity was observed for glycitein [52]. Positive effects against UV radiation were also noted for 6,7,4′-trihydroxyisoflavone, a major metabolite of daidzein, in solar-UV-induced normal human dermal fibroblasts. This compound acts as an inhibitor of protein kinase C (PKCα), which modulates the activity of matrix metalloproteinases (MMPs), enzymes that play a crucial role in collagen degradation in the skin [53].

The influence of fractions obtained from soybean extract containing different isoflavone derivatives, including aglycones (GIs), glucosides (GIIs), acetylglucosides (GIIIs), and malonylglucosides (GIVs), on the viability of UV-treated keratinocyte cells was tested by Chiang et al. [24]. They found that all tested extracts inhibited UVB-induced cell death; however, the acetylglucoside fraction showed the highest protective activity. The extracts also decreased UVB-induced intracellular H_2_O_2_ production. The investigation suggested that the MAPK pathway was involved in the action of isoflavones. UVB-induced JNK phosphorylation was strongly inhibited by group III and moderately inhibited by groups I and II, while no effect was observed for group IV. None of the isoflavone extracts affected UVB-induced ERK1/2 or p38 activation [24].

In another study, Chiu et al. investigated the efficacy of soybean extract (ISO-1) containing 12 isoflavones (total isoflavones: 43.8 µg/g; no data on the isoflavone profile). They observed that pre-incubation with ISO-1 decreased UVB-induced apoptosis in HaCaT cells. Furthermore, it also prevented the depletion of catalase, an enzyme involved in the neutralization of reactive oxygen species (ROS) produced in UV-irradiated cells. They also observed the activation of the MAPK signaling pathway; however, in contrast to Chiang et al., they noted a decrease in the phosphorylation of not only JNK but also p38 and ERK1/2 [25]. Moreover, Huang et al., who investigated different fractions isolated from soybean cake byproducts during the processing of soybean oil, found that the fraction containing aglycone and acetylglucoside forms of isoflavones (totaling 7.86 mg/g) inhibits UVB-induced apoptosis of human keratinocytes [54].

There are also a few in vivo studies regarding the photoprotective effects of soy isoflavones. An investigation of ICR-Foxn/nu mice revealed that an isoflavone mixture and genistein had an inhibitory effect on UVB-induced epidermal proliferation, suppressed the expression of COX-2 and PCNA, alleviated erythema, and reduced catalase depletion when the isoflavone extract was topically applied before UVB irradiation. It also reduced wrinkles and skin desquamation [25]. A further study by the research group, using a purified fraction containing isoflavone aglycones and their acetyl glucosides, confirmed the above activities [25]. The authors observed that the effectiveness of the extract fraction was higher than that of the mixture of 12 isoflavones; however, it should be noted that the concentration of the extracts used in both studies was different (3 mg/mL vs. 3–30 µg/mL, respectively).

The protective effect of topically applied genistein was noted by Wei et al., who found that pretreatment with 10 μM of genistein for 1 h prior to UVB exposure significantly inhibited damage to the epidermis and the internal organs of mice [55].

In addition, experiments conducted by Brand and Jendrzejewski [51] revealed that applying genistein to mouse skin before UV exposure reduced the number of apoptotic sunburn cells, although it did not impact leukocyte infiltration. In turn, when genistein was applied after irradiation, the reduction in apoptotic cells was insignificant, but it did decrease leukocyte numbers. Additionally, no effect on UV-induced epidermal hyperproliferation was observed with genistein. However, they found that applying genistein both before and after UV exposure prevented the disruption of intercellular adhesion proteins, such as E-cadherin, suggesting that it may help protect against the development of skin cancer [51]. The protective activity of genistein was also demonstrated in a rat model [50] and in the case of cutaneous changes induced by psoralen combined with UVA radiation in mice [56].

Another in vivo study showed that a fraction rich in isoflavone aglycones and acetylglucosides prevents photoaging by reducing desquamation, trans-epidermal water loss (TEWL), erythema, and epidermal thickness in mouse skin exposed to UVB irradiation. It also suppresses the expression of PCNA [54].

The experimental data and the observed effects of in vitro and in vivo studies are summarized in Table 2.

## 4. Anti-Inflammatory and Antioxidant Activity

Anti-inflammatory activity is important for maintaining healthy skin, as inflammation can lead to various skin disorders, including acne, psoriasis, and eczema. Chronic inflammation disrupts the skin’s barrier function, leading to increased sensitivity, redness, and irritation. By reducing inflammation, anti-inflammatory agents can help restore skin balance, alleviate discomfort, and promote healing.

The effects of isoflavones were tracked in cells stimulated with various factors, including TNF-α, lipopolysaccharides (LPS), IL-22, and IL-17A. The investigation focused on pro-inflammatory interleukins, such as IL-6, IL-8, IL-20, and IL-1β, as well as cyclooxygenase activity (COX-2), chemokine ligand 2 (CCL2), and transforming growth factor beta (TGF-β1). The role of pro-inflammatory interleukins in modulating inflammation is well-documented in the literature. These interleukins are crucial for triggering inflammatory responses by activating immune cells, enhancing the production of inflammatory mediators, and recruiting more immune cells to the site of inflammation. For instance, IL-1 and TNF-α induce fever, increase vascular permeability, and stimulate the production of acute-phase proteins. IL-6 promotes the acute-phase response and supports the differentiation of T and B cells, while IL-17 amplifies inflammation by inducing additional cytokine and chemokine production by recruiting neutrophils to the affected tissue. Overproduction of these interleukins can result in chronic inflammation and tissue damage. In turn, CCL2 plays a key role in recruiting monocytes, memory T cells, and dendritic cells to sites of inflammation. COX-2 contributes to the biosynthesis of prostaglandins, which modulate the inflammatory process. Meanwhile, TGF-β1 has a dual role in inflammation. It acts as an anti-inflammatory agent by suppressing immune responses and promoting tissue healing, but, in chronic inflammation, it can drive pro-inflammatory pathways and contribute to fibrosis.

Some in vivo and in vitro studies have demonstrated the effect of isoflavones on cytokines production in skin cells with induced inflammation. In a paper by Smolińska et al., the anti-inflammatory activity of genistein (100 μM) was assessed in normal keratinocytes and “psoriasis-like” keratinocytes (HaCaT cells stimulated with a cytokine mixture including IL-1A, IL-17A, IL-22, oncostatin M, and TNF-α, or LPS). Regardless of the inducer used, the expression of IL-8, IL-20, and CCL2 (but not IL-1B or TGF-β1) was observed, and genistein treatment decreased levels of these cytokines (with no changes for IL-1β or TGF-β1). Smolińska et al. also investigated the role of NF-κB signaling cascades in the modulation of anti-inflammatory activity [57]. They found that genistein prevented NF-κB translocation induced by the “cytokine mix” as well as TNF-α, and the inhibitory effect of genistein on the production of inflammatory cytokines was due to partially suppressing the ROS/NF-κB pathway. No impact on the PI3K signaling cascade was observed [57].

The anti-inflammatory activity of genistein was also noted by Li et al. in TNF-α-stimulated human synoviocyte MH7A cells (fibroblast-like cells) [26]. Genistein, in the range of 5–20 μM, decreased the levels of IL-1β, IL-6, and IL-8, suppressed the translocation of NF-κB, and inhibited the phosphorylation of IκB kinase-α/β and IκBα, which are responsible for the activation of NF-κB. They indicated that the inhibitory effect of genistein on cytokine production is a result of suppressing the ROS/Akt/NF-κB pathway and promoting adenosine monophosphate-activated protein kinase (AMPK) activation [26]. In addition to regulating cellular energy balance, AMPK also limits inflammation [58].

The inflammation-reducing properties of isoflavones were also demonstrated in an in vivo study. The Nova Soy isoflavone product, containing 33 mg of genistein and 67 mg of daidzein per 100 mg, inhibited 12-O-tetradecanoylphorbol-13-acetate (TPA)-induced cutaneous inflammation by modulating COX-2 and NF-κB in Swiss albino mice. It reduced edema formation, inhibited lipid peroxidation, and decreased NO production [59].

Soy isoflavones have also demonstrated an anti-psoriatic effect. Psoriasis is a long-lasting, recurring disorder characterized by excessive skin cell growth (hyperplasia), erythema, and scaling, affecting approximately 1–3% of the global population. Wang et al. demonstrated that a cream containing genistein (0.5% or 2%) significantly alleviated imiquimod (IMQ)-induced skin lesions in mice, reduced epidermal thickness, and suppressed the expression of inflammatory factors, including interleukin (IL)-1β, IL-6, tumor necrosis factor-alpha (TNF-α), chemokine ligand 2 (CCL2), IL-17, and IL-23. Genistein also inhibited TNF-α-induced proliferation of HaCaT cells and decreased the expression of IL-1β, IL-6, IL-8, IL-23, TNF-α, CCL2, and VEGFA. The authors suggested that the mechanism of action involved the STAT3–NF-κB pathway, as they observed inhibition of TNF-α-induced expression of phosphorylated STAT3 (pSTAT3), I-kBα (pI-kBα), and nuclear translocation of NF-κB [27]. A further in vivo study in mice found that the topical application of isoflavone extract prior to IMQ treatment reduced unfavorable effects, including trans-epidermal water loss (TEWL), erythema, and blood flow, while increasing surface skin hydration and attenuating epidermal hyperplasia and inflammatory cell infiltration [28]. Additionally, isoflavone extract reduced IL-22, IL-17A, and TNF-α-induced MAPK, NF-κB, and JAK-STAT pathway activation in normal human epidermal keratinocytes [28].

Because an excess of reactive oxygen species (ROS) is considered one of the main factors contributing to skin inflammation, antioxidant activity is often examined in the context of anti-inflammatory activity. Antioxidants protect skin cells from oxidative stress and support the skin’s repair processes by neutralizing excess free radicals caused by environmental factors, such as UV radiation. An accumulation of reactive oxygen species (ROS) can lead to premature aging, inflammation, and skin damage.

The antioxidant effect may occur through both direct scavenging activity and the modulation of the cellular redox state via its impact on antioxidant enzymes. The most common chemical tests used in these investigations include DPPH, ABTS, ORAC, and FRAP assays. These tests have demonstrated the antioxidant potential of soy isoflavones; however, it should be pointed out that their effectiveness is rather mild [60,61,62,63]. Furthermore, using cyclic voltammetry, it was found that daidzein (1.0 × 10^−4^ M) and its derivatives effectively retard lipid oxidation in liposomal membranes [2]. Similarly, genistein, at concentrations of 15 and 30 μM, inhibited lipid peroxidation and prevented lipid oxidation in both simple lipid systems (liposomes) and more complex lipoproteins by scavenging lipid peroxyl radicals [64].

The ROS scavenging activity of isoflavones was also examined in a cell-based model. Pretreatment with genistein (100 µM) attenuated the level of reactive oxygen species (ROS) in TNF-α and LPS-stimulated HaCaT cells [57]. Furthermore, similarly to 17β-estradiol, it decreases the ROS level and modulates eNOS/iNOS-dependent NO release and GSH content in human fibroblasts and keratinocytes under H_2_O_2_-induced oxidative stress. Additionally, it prevents H_2_O_2_-induced cytotoxicity, suppresses MMP-1 and MMP-9 expression, and prevents the decrease in mitochondrial membrane potential. Experimental data suggest that the mechanisms of action involve the p38 MAPK, Akt, and ERK1/2 signaling pathways [29]. On the other hand, daidzein, genistein, and glycitein at concentrations of 10 µg/mL demonstrated no ROS scavenging effects in skin fibroblasts and keratinocytes under oxidative stress conditions and did not affect the SOD, CAT, or GPH activity [39].

As mentioned in Section 3, isoflavones are able to reduce ROS in cells induced by UVB irradiation, which is one of the mechanisms of photoprotection [24,25,52]. An in vivo study also confirmed the effectiveness of these compounds in oxidative stress provoked by UVB. Wei et al. found that topical application of 10 μM of genistein on the skin of SKH-1 hairless mice decreased UVB-induced H_2_O_2_ levels by more than 50%. It also inhibited UVB-induced malondialdehyde (MDA) formation, a byproduct of lipid peroxidation, by approximately 30% to 50% [55].

Typically, in cell-based assays to monitor ROS, the 2′,7′-dichlorodihydrofluorescein diacetate (H_2_DCF-DA) method is employed. It is based on the oxidation of the non-fluorescent H_2_DCF-DA by intracellular ROS, which converts it into a highly fluorescent compound, 2′,7′-dichlorofluorescein (DCF). This fluorescence reflects ROS levels within the cells. However, Jurzak et al. used X-band electron paramagnetic resonance spectroscopy to examine the effect of genistein on ROS concentrations in normal and keloid fibroblasts exposed to UVB. They found that genistein (3.7 and 37 µM) altered the concentration of free radicals in both cell types [61].

The experimental data from in vitro and in vivo assays on the anti-inflammatory and antioxidant activity of soy isoflavones are summarized in Table 3.

## 5. Anti-Aging Effects

Skin aging is a physiological process. With the passage of time, the skin undergoes degenerative changes, including decreased elasticity, reductions in epidermal thickness and collagen content, elastic fiber degeneration, and increased dryness, all of which accelerate the aging process and contribute to wrinkle development. It is commonly known that aging can be significantly delayed by the administration of estrogen and compounds with estrogen-like activity. Therefore, isoflavones, which belong to phytoestrogens, seem to be ideal candidates for anti-aging formulations [65,66]. Many reports indicate that oral administration of soy isoflavones improves skin condition and promotes skin repair [67,68,69,70,71]. Additionally, there are studies that have examined these effects during topical application of soy isoflavones.

It is known that extracellular matrix (ECM) components, including collagen and elastic fibers, which are responsible for the structural and elastic qualities of the skin, decrease with age. Mi-Sun et al. found that daidzein, similarly to β-estradiol, significantly increases the mRNA expression of collagen types I and IV, elastin, and fibrillin-1 in normal human dermal fibroblasts (NHDFs) [72]. Zhao et al. also observed that daidzein treatment increases collagen synthesis and inhibits collagen degradation in daidzein-treated fibroblasts and mouse skin. This was accompanied by the suppression of metalloproteinases MMP-1 and MMP-2, which are responsible for breaking down extracellular matrix (ECM) components, such as collagen and elastin. Because they observed elevated levels of transforming growth factor (TGF-β), a cytokine that plays a crucial role in regulating collagen synthesis, along with phosphorylated Smad2 and Smad3 (the immediate downstream targets of TGF-β1), it can be concluded that the TGF-β/Smad signaling pathways are involved in daidzein-induced collagen accumulation [35]. Similar effects were noted in the case of fibroblasts treated with glycitin, which included elevated synthesis of collagen types I and III, increased levels of fibronectin and TGF-β, enhanced phosphorylation of Smad2, Smad3, and AKT, and decreased levels of MMP-1 [73].

Furthermore, Sienkiewicz et al. investigated the impact of genistein on collagen biosynthesis in normal human dermal fibroblasts (CRL-1474) subjected to oxidative stress induced by t-butylhydroperoxide (t-BHP). The results showed that the effect is highly concentration-dependent; at 1 μM, genistein prevents the inhibition of collagen biosynthesis, while at 10 μM, the protective effect diminishes, and at 100 μM, it actually enhances the inhibition. They found that the protective effect of genistein against oxidative stress may result from the prevention of disruptions in the IGF-I receptor-mediated signaling pathway, which is associated with ERK1/ERK2 and can be triggered by oxidative agents [74]. It has also been shown that genistein has a modulatory effect on the expression of AP-1 subunits C-JUN, C-FOS, and FOS-B in skin keratinocytes, fibroblasts, and keloid fibroblasts. Because AP-1 is associated with cell proliferation, differentiation, and apoptosis, as well as ECM synthesis, this indicates that genistein may have potential as an anti-aging agent [75]. In addition, daidzein and genistein increase hyaluronic acid production in human keratinocytes [76].

A beneficial effect in the context of anti-aging activity has also been demonstrated for soy extracts containing a mixture of isoflavones. Leaf extract enriched in isoflavones increased collagen levels by inducing the expression of genes related to its synthesis, COL1A1 and COL3A1, in human dermal fibroblasts [77]. In another study, soy extract stimulated the production of type I procollagen in fibroblast cultures [78].

In vivo studies have also indicated the anti-aging potential of isoflavones or soy-based formulations containing isoflavones. It has been evidenced that 24 weeks of treatment with 4% genistein gel enhances hyaluronic acid concentration in postmenopausal skin [79]. Furthermore, it led to improvements in skin parameters, including epidermal thickness and the number of blood vessels. However, it should be noted that the effect was significantly weaker compared to estradiol [80]. Additionally, a double-blind, placebo-controlled study using 2% soy extract in 21 volunteers showed that the topical application of an emulsion with isoflavones significantly increased the number of dermal papillae, thereby rejuvenating the dermal–epidermal junction in aging skin by enhancing the interlocking between the epidermis and dermis [81].

Moreover, an animal study showed that commercial soy serum improves morphometric parameters, including the total epidermal width, the keratinocyte nuclear volume, and the proliferating cell nuclear antigen index in the basal, spinous, and peripheral layers of the epidermis [82]. Additionally, bifidobacterium-fermented soy milk extract (BE) containing genistein and daidzein increased the elasticity and hyaluronic acid content while enhancing skin hydration [83]. The isoflavone fraction also demonstrated a protective effect against photoaging, as it decreased desquamation, trans-epidermal water loss, erythema, and epidermal thickness in mouse skin exposed to UVB irradiation [54].

Table 4 displays the experimental details for in vitro and in vivo assays on the anti-aging effects of soy isoflavones.

## 6. Other Effects of Isoflavones on the Skin

Besides their anti-inflammatory, protective, and anti-aging properties, various other beneficial effects of soy isoflavones have also been reported. Isoflavones, particularly genistein and genistin, have also been examined in the context of anti-melanoma activity. Melanoma is an aggressive form of skin cancer that develops in melanocytes, the cells responsible for producing melanin. Certain factors, such as excessive UV exposure and sunburns, increase the risk of developing melanoma. The study by Russo et al. demonstrated that genistin significantly reduced the viability of melanoma cells after 3 days of exposure. Furthermore, genistin induced DNA strand breaks. Notably, in contrast to normal cells, where isoflavones decrease intracellular ROS levels, genistin significantly increased intracellular ROS levels in melanoma cells [84]. A similar effect of various flavonoids on ROS in cancer cells has been reported in the existing literature. It has been found that cancer cells exhibit impaired antioxidant capacity compared to normal cells due to decreased catalase activity [40]. Interestingly, daidzein did not have a negative effect on melanoma cells, suggesting that the number of hydroxyl groups determines the effectiveness of isoflavones [84]. The anti-melanoma mechanism of action of isoflavones is associated with their impact on the cell cycle and cyclin-dependent kinases (CDKs). It has been found that genistein (30 µM) arrests cells in the G2 phase by inhibiting CDK1 by 50–70%, while daidzein (150 µM) induces an accumulation of cells in the G1 phase by inhibiting CDK2 by 40–60% [37]. Furthermore, genistein alters the shape of melanoma cells and the cytoskeletal network [85].

Soy isoflavones have also been investigated for their potential in wound healing. In vitro assays showed that glycitein (10 and 20 µM) significantly induced the proliferation and migration of skin fibroblasts [73]. Additionally, a stimulatory effect on keratinocyte proliferation was observed with an enzymatically hydrolyzed soy isoflavone fraction, indicating the potential of isoflavones in promoting skin regeneration during wound healing [86]. Furthermore, it has been demonstrated that nanofiber wound dressings made from soy protein isolate combined with genistein and hyaluronic acid tested in ovariectomized mice and ex vivo human skin tissues promote cutaneous tissue repair via the ER-β signaling pathways [87].

Genistein may also have a protective effect against scar formation. Cao et al. found that it inhibits cell proliferation and collagen synthesis in fibroblasts from hypertrophic scars [88]. Moreover, it has been shown that genistein treatment effectively reduces retinoid-induced epidermal hyperplasia and suppresses the rapid proliferation of keratinocytes in a monolayer culture [78].

The aforementioned effects of isoflavones on skin cells are summarized in Table 5.

## 7. Conclusions and Future Directions

Research on phytoestrogens has expanded significantly in recent years, as evidenced by numerous publications. This review aims to compile data on the anti-inflammatory, protective, and anti-aging activities of soy isoflavones, supporting their potential for topical application. This information may assist researchers in designing future studies.

Our investigation reveals that despite the widespread incorporation of isoflavones, particularly genistein, into cosmetic products, understanding of their effects on skin cells and the molecular mechanisms underlying their efficacy remains limited. Although several studies have examined the in vitro and in vivo effects of phytoestrogens, many questions remain unresolved. It should also be noted that the majority of research focuses on genistein, while knowledge about other isoflavones remains scarce. Due to the structural diversity among soy isoflavones, their biological activities vary, and additional, as-yet-unstudied effects may exist. Further studies using diverse animal models are also needed to assess the toxicity, pharmacokinetics, pharmacodynamics, and bioavailability of isoflavones. Currently, most research has focused on the in vitro effects of isoflavones, highlighting the need for more in vivo and clinical studies to fully evaluate their efficacy and safety for skin applications.

In summary, despite the promising potential demonstrated, further research is essential to elucidate the mechanisms of action of isoflavones, optimize treatment protocols, and clarify their specific therapeutic effects. Future research should focus on advancing our understanding of the specific mechanisms of action of different isoflavones, as well as expanding studies to explore new directions associated with their health-promoting effects on the skin. Additionally, more in vivo and clinical studies, which are crucial for comprehensively assessing the safety and therapeutic impact of these compounds, should be planned to clarify their pharmacokinetics, pharmacodynamics, and potential toxicity. Efforts should also be made to enhance their bioavailability for more effective use. Developing and refining nanotechnology-based delivery systems that could improve the solubility, permeability, and bioavailability of isoflavones, thereby enhancing their potential efficacy in topical applications, is another important direction for further investigation.

## Figures and Tables

**Table 1:** The effect of 2 h pretreatment with isoflavones on DNA damage and cyclooxygenase-2 (COX-2) in BJ-5ta cells. The cells were harvested 24 h after irradiation (60 mJ/cm^2^).

Compounds (Concentrations Tested)	Observed Effect	Ref.
Cyclooxygenase-2 (COX-2)		
genistein (Gen) (2, 4, 6, 10 µM)	↑ expression: 2, 4, 6 μM	[47]
daidzein (Dai) (8, 16, 24, 30 µM)	↑ expression COX-2 (8 μM)	[47]
mix of Gen:Dai (2:8; 4:16; 6:24; 10:30 μM)	↓ expression: 2:8, 4:16, 6:24 μM ↑ expression: 10:30 μM	[47]
genistein (GenG) (2, 4, 6, 8, 10, 20, 30, 60 µM)	↓ expression for all concentrations	[48]
daidzein (DaiG) (2, 4, 6, 8, 10, 20, 30, 60 µM)	↓ expression: 2, 4, 8 μM	[48]
mix of GenG:DaiD (2:2; 2:4; 4:8; 6:8; 8:8; 8:2; 10:4; 10:10 µM)	↓ expression: 6:8 μM; 10:10 μM ↑ expression 10:4 μM	[48]
RPH–aglycone mix * (2, 4, 6, 8, 10, 20, 30 µM)	↓ expression COX-2: 8, 10 μM	[48]
DNA damage (comet assay)		
genistein (Gen) (10, 30, 60 µM)	↓ tail moment: 30, 60 µM	[47]
daidzein (Dai) (10, 30, 60 µM)	↓ tail moment: 10, 30, 60 µM	[47]
mix of Gen:Dai (2:8; 4:16; 6:24 μM)	↓ tail moment (2:8, 4:16, 6:24 μM)	[47]
genistein (GenG) (2, 6, 10 µM)	↑ tail moment for all concentrations	[48]
daidzein (DaiG) (2, 6, 10 µM)	↑ tail moment for all concentrations	[48]
mix of GenG:DaiD (2:2; 2:10; 4:10; 6:8 µM)	↓ tail moment: 2:2 µM	[48]
RPH–aglycone mix * (2, 4, 6, 8, 10, 20, 30 µM)	↓ tail moment (8, 10 µM)	[48]
DNA damage (Gadd45 expression)		
genistein (Gen) (2, 4, 6, 10 µM)	↑ expression: 2, 6 μM	[47]
daidzein (Dai) (8, 16, 24, 30 µM)	↑ expression: 8, 16 μM	[47]
mix of Gen:Dai (2:8; 4:16; 6:24; 10:30 μM)	↓ expression: 2:8, 4:16, 6:24 μM ↑ expression: 10:30 μM	[47]
genistein (GenG) (2, 4, 6, 8, 10, 20, 30, 60 µM)	↑ expression: 2, 6, 10, 30, 60 μM	[48]
daidzein (DaiG) (2, 4, 6, 8, 10, 20, 30, 60 µM)	↓ expression: 2, 4 μM ↑ expression: 10–60 μM	[48]
mix of GenG:DaiD (2:2; 2:4; 4:8; 6:8; 8:8; 8:2; 10:4; 10:10 µM)	↓ expression (2:2 μM) ↑ expression (the other mix)	[48]
RPH–aglycone mix * (2, 4, 6, 8, 10, 20, 30 µM)	↑ expression for all concentrations	[48]

* soy extract titrated to 90% in isoflavone aglycones; ↑—increase; ↓—decrease.

**Table 2:** The experimental data from in vitro and in vivo assays on the photoprotective activity of soy isoflavones.

Component/Treatment	Model	Observed Effects	Ref.
Genistein (10, 20, 50 µM) added 1 h prior to UVB	EpiDermFT Full Thickness (skin model comprising NHK)/UVB 20, 60 mJ/cm^2^)	↓ UV induced PD formation Preservation of PCNA expression	[49]
Genistein (5 µM) added 24 h prior to UVB	HaCaT/UVB 50 mJ/cm^2^	↓ UV induced IL-1, MIF, PLANH1 CXCL1; IL-8—no effect	[50]
Gen, Dai, Glycitin (10 mM) Mix (55% Gen, 43% Dai, 1.8% Gly) (10 mM) added 12 h prior to UVB	HaCaT/UVB 70 mJ/cm^2^	↓ UV induced H_2_O_2_ (no effect for glycitine)	[52]
Fraction (0.5, 1%): G1-aglycone, G2-glucoside, G3-acetylglucoside, G4-malonylglucoside added prior to UVB and incubated for 24 h	HaCaT/UVB 40 mJ/cm^2^	↑ Viability of UVB-irradiated cell (G3 most effective) ↓ UVB-induced intracellular H_2_O_2_ ↓ UVB induced phosphorylation of JNK (no effect on ERK1/2 or p38)	[24]
Extract with 12 isoflavones (total Iso: 43.8 µg/g) added prior to UVB and incubated for 24 h	HaCaT/UVB 50 mJ/cm^2^	↑ Viability of UVB-irradiated cell ↓ UVB induced phosphorylation of ERK1/2, JNK, p38 Prevention of the depletion of catalase	[25]
Extract with Gen, Dai, Gly, and their acetyl glucosides (total Iso: 7.86 mg/g) added 24 h prior to UVB	HaCaT/UVB 50 mJ/cm^2^	↑ Viability of UVB-irradiated cell ↓ UVB induced phosphorylation of ERK1/2, JNK, p38	[54]
Genistein (10 μM) applied 1 h prior to UVB	SKH-1 hairless mouse skin/UVB 0.15-15 kJ/m^2^	↓ UVB induced H_2_O_2_ ↓ UVB induced MDA ↓ Indicators of DNA damage (8-OHdG, TD)	[55]
Genistein (0.5, 2 nmol/cm^2^) applied 1 h prior to UVA	SKH-1 mouse skin/8-methoxy-psolaren and UVA	↓ UV induced the epidermal thickness	[56]
Genistein (0.1%, 1%) applied 24 h prior to UVB	Rat model/UVB 50 mJ/cm^2^	Prevent wrinkle formation	[50]
Genistein (5 μM) applied 1 h prior to UVB 1 or 4 h after UVB	SKH-1 hairless mouse skin/UVB 60 mJ/cm^2^	↓ Sunburned cells (Gen prior to UVB exposure) ↓ Leukocyte infiltration (Gen after UVB exposure) No effect on epidermal hyperproliferation Restoration of E-cadherin levels	[51]
Extract with 12 isoflavones (total Iso: 43.8 µg/g); genistein (1, 3 mg/mL) applied prior to UVB for 7 days	Mouse dorsal skin/UVB 150 mJ/cm^2^	↓ The epidermal thickness ↓ Erythema ↓ TEWL ↓ Desquamation ↓ UVB-induced H_2_O_2_ levels ↓ COX-2 ↓ PCNA expression Prevention of catalase depletion	[25]
Extract with Gen, Dai, Gly, and their acetyl glucosides (7.86 mg/g) applied prior to UVB for 7 days	Mouse dorsal skin/UVB 150 mJ/cm^2^	↓ The epidermal thickness ↓ Erythema ↓ TEWL ↓ Desquamation	[54]

NHK—normal human keratinocytes; PD—pyrimidine dimers; 8-OHdG—8-hydroxy-2′-deoxyguanosine; MDA—malondialdehyde; TD—thymine dimmers; CXCL1—chemokine (C-X-C motif) ligand 1; PCNA—proliferating cell nuclear antigen; IL—interleukin; MIF—macrophage migration inhibitory factor; PLANH1—plasminogen activator inhibitor; TEWL—trans-epidermal water loss; COX-2—cyclooxygenase 2; ↑—increase; ↓—decrease.

**Table 3:** The experimental data from in vitro and in vivo assays on the anti-inflammatory and antioxidant activity of soy isoflavones.

Compound/Extract	Model	Effect	Ref.
Genistein 50, 10 µM	TNF-α-treated HaCaT	↓ IL-1β, IL-6, IL-8, IL-23, TNF-α, VEGFA, MCP1 ↓ TNF-α-induced proliferation	[27]
Genistein 20 μM	TNF-α-stimulated human synoviocytes (MH7A)	↓ IL-1β, IL-6, and IL-8 ↓ ROS	[26]
Genistein 100 μM	Normal HaCaT, “psoriasis-like” HaCaT *	↓ IL-8, IL-20, and CCL2 ↓ ROS No changes in IL-1β or TGF-β1	[57]
Genistein 1, 10, 100 μM	Human fibroblasts and keratinocytes induced with H_2_O_2_	↓ NO, ↓ ROS, ↑ GSH Prevents H_2_O_2_-induced cytotoxicity ↑ Mitochondrial membrane potential ↓ MMP-1, MMP-9	[29]
Nova Soy containing 33 mg of Gen, 67 mg of Dai/100 mg	Swiss albino mice with TPA-induced cutaneous inflammation	↓ COX-2, ↓ NO ↓ MDA (lipid peroxidation) ↓ Edema Restoration of SOD, CAT, GSH ↓ TNF-α, IL-6, IL-1 β	[59]
Isoflavone extract **	NHEK induced with IL-22, IL-17A, TNF-α	↓ Phosphorylation of STAT3, JAK2, ERK, JNK, and p38	[28]
Cream containing genistein (0.5% or 2%)	Imiquimod (IMQ)-induced skin lesions in mice	↓ IL-1β, IL-6, IL-17, IL-23, TNF-α, CCL2 Reduction of epidermal thickness	[27]
Isoflavone extract **	Imiquimod (IMQ)-induced skin lesions in mice	↓ TEWL, erythema, blood flow, thickness Attenuation of hyperplasia and cell infiltration	[28]

* HaCaT cells stimulated with a cytokine mixture including IL-1A, IL-17A, IL-22, oncostatin M, and TNF-α; NHEK—normal human epidermal keratinocyte; TEWL—trans-epidermal water loss blood; GSH—glutathione; SOD—superoxide dismutase; CAT—catalase, COX-2—cyclooxygenase 2; MDA—malondialdehyde; CCL2—chemokine ligand 2; MMP—matrix metalloproteinase; TGF—transforming growth factor; TPA—12-O-tetradecanoylphorbol-13-acetate; **—no data on chemical composition; ↑—increase; ↓—decrease.

**Table 4:** The experimental data from in vitro and in vivo assays on the anti-aging effects of soy isoflavones.

Compound/Extract	Model	Effect	Ref.
Daidzein (0.1, 1, 10 μg/mL)	Normal human dermal fibroblasts	↑ Collagen type I and IV ↑ Elastin, fibrillin-1	[72]
Daidzein (0.5, 5, 50 μg/mL)	Human skin fibroblasts	↓ MMP1 and 2 ↑ Collagen type I ↑ TGF-β ↑ Phosphorylated Smad2 and 3	[35]
Glycitin (20 μM)	Human skin fibroblasts	↓ MMP1, ↑ collagen type I, III ↑ TGF-β ↑ fibronectin ↑ Phosphorylated Smad2 and 3 and AKT	[73]
Genistein (4 × 10^−9^–4 × 10^−7^ M) Daidzein (4 × 10^−7^ M)	Normal human keratinocytes	↑ Hyaluronic acid	[76]
Genistein, daidzein (100 ng/mL)	Human dermal fibroblasts	↑ Collagen for genistein No effect for daidzein	[81]
Genistein (1, 10, 100 μM)	Fibroblasts induced with t-BHP	↑ Collagen (1 μM) ↓ collagen (10, 100 μM) Protection of DNA biosynthesis (1, 10 μM)	[74]
Soy powder (250 mg/2 mL) (40, 200 μg/mL)	Human dermal fibroblasts	↑ Type I procollagen	[78]
Daidzein (200 μg/mL 6 weeks)	BALB/C mice	↑ Type I collagen	[35]
Gen (4 × 10^−5^–4 × 10^−4^ M) Dai (2 × 10^−4^–4 × 10^−4^ M) (2 weeks 0.1 mL/5 cm^2^/day)	Hairless mice	↑ Hyaluronic acid	[76]
4% genistein gel (24 weeks)	Postmenopausal women	↑ Hyaluronic acid ↑ Epidermal thickness ↑ Number of blood vessels	[79,80]

t-BHP—t-butylhydroperoxide; MMP—matrix metalloproteinase; TGF—transforming growth factor; ↑—increase; ↓—decrease.

**Table 5:** The other effects of soy isoflavones.

Type of Activity	Compound/Model	Effect	Ref.
Wound healing	Glicitin (0.5, 1, 2, 5, 10, 20 µM)/human dermal fibroblasts	↑ Cell proliferation (10, 20 µM) ↑ Cell migration (10, 20 µM)	[73]
	Isoflavone-aglycone-rich extracts/human keratinocytes	↑ Cell proliferation	[86]
Anti-melanoma	Genistin (GenG), daidzin (DaiG) (12, 25, 50, and 100 µM)/skin melanoma cells	↓ Cell viability (59.1% for 100 µM GenG; no effect for DaiG) ↑ ROS (50, 100 µM)—DaiD not studied ↑ DNA damage (50, 100 µM)—DaiD not studied	[84]
	Genistein (5, 10, 20, 30 µM)/skin melanoma cells	↓ Cell viability (IC_50_ 15.5 μM) Changes in cell shape and cytoskeleton ↑ Melanin content ↑ Tyrosinase activity	[85]
	Genistein/skin melanoma cells	↓ Cell viability (IC_50_ after 24 h 200 μM; IC_50_ after 48 h 35 μM; IC_50_ after 5 days 12.5 μM)	[89]
	Genistein (30, 50, and 80 μmol/L)/skin melanoma cells	↓ Basal and PGE2-stimulated cell proliferation	[90]
	Genistein 100, 150, 200 μM)/skin melanoma cells	↓ Protein tyrosine phosphorylation ↓ Cells to invade through ECM	[91]
	Genistein (0.5, 1, 5, 10, 50, 100 μM)/skin melanoma cells	IC_50_ 6.6 μM after 96 h of exposure (no cytotoxic effects for 24 h treatment) ↑ Adhesion of cells (0.5 to 50 μM) ↓ Cell migration (20 to 50 μM)	[92]
Anti-scarring	Genistein (25, 50, 100 µM)/hypertrophic scar specimens	↓ Collagen synthesis (50, 100 µM) ↓ Fibroblast proliferation 50, 100 µM (after 24 h) 25, 50, 10 µM (after 48 and 72 h)	[88]
Anti-hyperplasia	Genistein (0.5, 1 μg/mL)/ retinoid-induced cultured skin	↓ Epidermal thickness Daidzein and glycitein—no effect	[78]

ECM—extracellular matrix; PGE2—prostaglandin E2; ↑—increase; ↓—decrease.

## Data Availability

Data are contained within the article.
